# FRY Mediates THP1-Driven Ovarian Cancer Invasion Through the PI3K/AKT Pathway

**DOI:** 10.3390/cells15030289

**Published:** 2026-02-03

**Authors:** Jin-Hyung Kim, Minjun Choi, Jae-Yoon Kim, Soo-Yeon Woo, Woo Yeon Hwang, Jung-Hye Choi

**Affiliations:** 1 Department of Biomedical and Pharmaceutical Science, College of Pharmacy, Kyung Hee University, Seoul 02447, Republic of Korea; jenon@khu.ac.kr (J.-H.K.);; 2 Institute of Integrated Pharmaceutical Sciences, College of Pharmacy, Kyung Hee University, Seoul 02447, Republic of Korea; 3 Department of Obstetrics & Gynecology, College of Medicine, Kyung Hee University Medical Center, Kyung Hee University, Seoul 02447, Republic of Korea

**Keywords:** FRY, ovarian cancer, tumor-associated macrophage, invasion

## Abstract

Ovarian cancer remains the most lethal gynecological malignancy, largely due to its early dissemination and extensive peritoneal metastasis. The tumor microenvironment (TME), particularly tumor-associated macrophages, promotes this invasive phenotype; however, the precise molecular effectors linking immune-to-tumor signaling remain unclear. We identified FRY, a microtubule-binding protein previously uncharacterized in ovarian pathology, as a critical mediator of macrophage-driven invasion. We observed that conditioned medium from ovarian cancer-stimulated macrophages (OCM) robustly induced FRY expression in ovarian cancer cells. Clinically, elevated FRY levels correlate with advanced tumor stage and poor patient survival. Functionally, FRY knockdown significantly abrogated OCM-induced invasion without affecting cell viability, highlighting its specific role in motility. Mechanistically, FRY facilitates epithelial–mesenchymal transition (EMT) and acts as an essential downstream effector of the PI3K/AKT signaling cascade; notably, FRY was required for AKT1-driven invasive behaviors. Furthermore, we identified the transcription factor NFIX as a key regulator of FRY expression. Macrophage-derived signals upregulate NFIX, which directly regulates FRY transcription. Pharmacological inhibition of the CXCR1/2 axis with reparixin effectively blocked OCM-mediated induction of both NFIX and FRY, suggesting that chemokine signaling initiates this pro-invasive loop. Collectively, these findings suggest that FRY is a macrophage-driven mediator of invasion and underscore its potential relevance in ovarian cancer.

## 1. Introduction

Ovarian cancer is one of the most lethal gynecological malignancies, with approximately 324,398 new cases and 206,839 deaths reported worldwide by 2022 [1]. Risk factors include age, family history, hereditary cancer syndromes, and mutations in BRCA1/2 [2]. Most patients are diagnosed at advanced stages due to subtle early symptoms and a lack of effective diagnostic tools. While early-stage disease shows favorable outcomes (stage I: ~90% cure rate; stage II: ~70% 5-year survival), advanced-stage patients (stage III–IV) face a significantly lower survival rates (≤20%), yet account for approximately 80% of diagnoses [3]. This poor prognosis necessitates a full elucidation of the underlying molecular pathways that drive the metastatic progression of ovarian carcinoma.

The tumor microenvironment (TME) significantly influences ovarian cancer progression by modulating tumor growth, metastasis, and therapeutic resistance. Within the TME, tumor-associated macrophages stand out as pivotal drivers of cancer cell invasion [4]. Macrophages originate from circulating peripheral blood monocytes and infiltrate tumor sites where they actively fuel metastasis through various strategies [5]. These strategies include the release of pro-invasive signaling molecules, such as cytokines and chemokines, upregulation of epithelial–mesenchymal transition (EMT), and promotion of peritoneal cancer cell adhesion [6,7,8]. Despite extensive research on the pro-metastatic functions of macrophages in the TME, the specific molecular pathways and gene expression changes that drive macrophage-induced invasion of ovarian cancer remain incompletely understood.

Microtubule-associated proteins (MAPs) play essential roles in regulating cellular processes critical for cancer progression, including cell division, migration, and invasion [9,10]. FRY (furry) is located on chromosome 13 and encodes a cytoplasmic microtubule-binding protein [11]. A recent study examined FRY in the context of breast cancer biology [11]; however, its broader functional significance remains unclear, and FRY has not been characterized in ovarian cancer. In our previous transcriptomic analysis of macrophage-stimulated ovarian cancer cells [12], FRY was identified as a gene highly upregulated in response to macrophage stimulation. This finding prompted us to investigate FRY’s functionality in ovarian cancer invasion, especially in response to macrophage-derived signals, and the underlying molecular mechanism.

## 2. Materials and Methods

### 2.1. Reagents and Materials

Culture media (RPMI 1640), antibiotics (penicillin, streptomycin), and FBS were obtained from WELGENE Inc. (Gyeongsan, Republic of Korea) and Hyclone (Logan, UT, USA), respectively. FRY siRNAs and gene-specific primers (Table S1) were synthesized by Bioneer Corporation (Seoul, Republic of Korea). AKT1 plasmids were obtained from Addgene (Cambridge, MA, USA), reparixin from MedChemExpress (Monmouth Junction, NJ, USA), and MTT reagent from Thermo Fisher Scientific (Waltham, MA, USA).

### 2.2. Cell Culture

Human ovarian adenocarcinoma cell lines (A2780, SKOV3, and ES2) and the human monocytic THP1 cell line were obtained from the American Type Culture Collection (ATCC; Manassas, VA, USA). All cell lines were routinely maintained in Roswell Park Memorial Institute (RPMI) 1640 medium (WELGENE Inc., Gyeongsan, Republic of Korea) supplemented with 5–10% fetal bovine serum (FBS), penicillin (100 U/mL), and streptomycin (100 μg/mL). Cells were cultured at 37 °C in a humidified incubator containing 5% CO_2_. THP1 cells were maintained in RPMI 1640 medium additionally supplemented with 0.05 mM β-mercaptoethanol. For macrophage differentiation, THP1 monocytes were seeded and treated with 100 nM phorbol 12-myristate 13-acetate (PMA; Sigma-Aldrich, St. Louis, MO, USA) for 24 h, resulting in adherent macrophage-like cells. After differentiation, PMA-containing medium was removed, and cells were washed with phosphate-buffered saline (PBS) prior to further conditioning. To generate ovarian cancer-conditioned macrophage medium (OCM), a two-step conditioning protocol was employed. Ovarian cancer cells were seeded at a density of 1.8 × 10^5^ cells in 100 mm culture dishes containing 10 mL of fresh complete RPMI medium and cultured for 24 h to obtain ovarian cancer cell-conditioned medium (CM). The collected CM was centrifuged at 2500 rpm for 3 min to remove cellular debris, and the supernatant was transferred to fresh tubes. PMA-differentiated THP1 macrophage-like cells were then incubated with ovarian cancer CM for an additional 24 h to induce ovarian cancer-conditioned macrophages. Following conditioning, the medium was replaced with fresh complete medium, and cells were cultured for another 24 h. The resulting OCM was collected, centrifuged at 2500 rpm for 3 min, aliquoted, and stored at −80 °C until further use.

### 2.3. Invasion Analysis

Cell invasion assays were performed using Matrigel-precoated Transwell chambers with 8 µm pore-size polycarbonate membranes (BD Biosciences, San Jose, CA, USA). Briefly, the upper surface of the Transwell inserts was coated with diluted Matrigel basement membrane matrix according to the manufacturer’s instructions and allowed to polymerize at 37 °C. Cells were resuspended in RPMI 1640 medium containing 1% FBS and seeded into the upper chambers, while RPMI 1640 medium supplemented with 5% FBS was added to the lower chambers as a chemoattractant. Following incubation for 48 h at 37 °C in a humidified atmosphere with 5% CO_2_, non-invading cells on the upper surface of the membrane were removed using cotton swabs. Invaded cells on the lower surface were fixed and stained with 5% (*w*/*v*) crystal violet solution. Images were acquired using an inverted microscope (Olympus, Tokyo, Japan), and invaded cells were quantified using ImageJ software (National Institutes of Health, Bethesda, MD, USA; version 1.53t).

### 2.4. Cell Viability Determination

Cell viability was assessed using the MTT assay. Cells were seeded into 96-well plates and treated as indicated. After incubation, MTT reagent was added to each well and incubated for an additional period to allow for formazan crystal formation. The resulting crystals were dissolved, and absorbance was measured at 540 nm using a SpectraMax spectrophotometer (Molecular Devices, Sunnyvale, CA, USA).

### 2.5. Clinical Data and Pathway Analyses

Publicly available ovarian cancer transcriptomic datasets were obtained from The Cancer Genome Atlas ovarian cancer cohort (TCGA-OV) [13] and the Gene Expression Omnibus (GEO) database (GSE9899, GSE63885) [14,15]. FRY expression levels and their association with clinical outcomes were analyzed using normalized gene expression data.

Overall survival analyses were performed using the Kaplan–Meier Plotter web tool (https://kmplot.com/; accessed on 6 May 2025), including a total of 1435 ovarian cancer patients. Patients were stratified into high- and low-expression groups based on the median FRY expression value. Hazard ratios (HRs), 95% confidence intervals (CIs), and log-rank *p*-values were calculated to assess statistical significance. Immune cell composition was estimated using CIBERSORTx based on bulk RNA-seq data from the TCGA-OV and GSE9899 cohorts [16]. Tumors were stratified into FRY-low and FRY-high groups according to median FRY expression, and relative proportions of immune cell subsets were compared between groups.

Gene Set Enrichment Analysis (GSEA) was conducted using the Molecular Signatures Database (MSigDB) HALLMARK gene sets. Enrichment significance was determined using a nominal *p*-value threshold of <0.05.

### 2.6. Gene Expression Quantification

Total RNA isolation, cDNA synthesis, and reverse transcription polymerase chain reaction (RT-PCR) were performed using reagents from Intron Biotechnology (Seoul, Republic of Korea), Enzynomics (Daejeon, Republic of Korea), and Takara (Kyoto, Japan), respectively. Target normalization used β-actin via the 2^−ΔΔCt^ method.

### 2.7. Western Blot

The cells were washed with cold PBS and lysed in protein lysis buffer (Intron Biotechnology) supplemented with protease and phosphatase inhibitors. Protein concentrations were measured using Bradford assay. Equal amounts of protein were mixed with 5× SDS sample buffer, boiled for 5 min, separated by polyacrylamide gel electrophoresis (SDS–PAGE), and transferred onto PVDF membranes. After blocking with 5% skim milk, the membranes were incubated with primary antibodies against β-actin (sc-47778), SNAI1 (sc-271977), and ZEB1 (sc-515797) (all from Santa Cruz Biotechnology, Dallas, TX, USA), and E-cadherin (3195S; Cell Signaling Technology, Danvers, MA, USA), followed by incubation with horseradish peroxidase-conjugated secondary antibodies. Signals were detected by chemiluminescence (Abclonal, Seoul, Republic of Korea) and quantified using ImageJ software.

### 2.8. Statistical Analysis

Statistical comparisons were performed using the Student’s *t*-test in GraphPad Prism 8 (GraphPad, San Diego, CA, USA), with *p* < 0.05 defining significance.

## 3. Results

### 3.1. FRY Is Upregulated by Macrophage Stimulation and Correlates with Poor Clinical Outcomes

**Figure 1:**
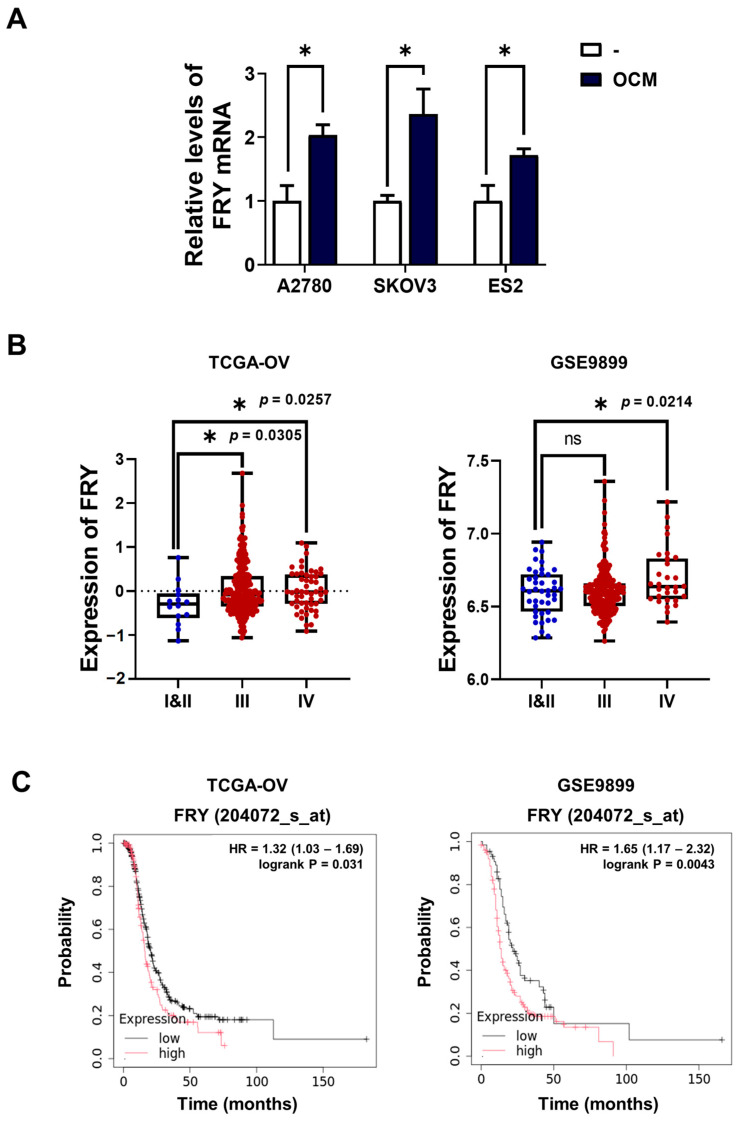
Macrophage-induced FRY upregulation and clinical significance in ovarian cancer. (**A**) RT-PCR analysis of FRY mRNA levels in A2780, SKOV3, and ES2 cells following 24 h stimulation with conditioned medium from ovarian cancer-stimulated macrophages (OCM). (**B**) FRY transcript levels across clinical stages in the TCGA-OV (stage I–II: *n* = 14; III: *n* = 248; IV: *n* = 53) and GSE9899 (stage I–II: *n* = 42; III: *n* = 219; IV: *n* = 30) cohorts. (**C**) Kaplan–Meier survival analysis showing reduced overall survival in patients with high FRY expression. * *p* < 0.05; ns, not significant.

Tumor-associated macrophages secrete diverse soluble factors that drive the reprogramming of cancer cells to a metastatic phenotype [17,18,19]. We hypothesized that macrophage-derived signals alter the gene expression patterns in ovarian cancer cells to promote invasion. Our previous transcriptomic analysis of ovarian cancer cells exposed to macrophage-CM identified multiple differentially expressed genes [12]. Among the up-regulated genes, we identified FRY as a potential mediator of macrophage-induced invasion. RT-PCR validation confirmed consistent FRY mRNA upregulation following OCM treatment in all tested cell lines (A2780, SKOV3, and ES2) (Figure 1A).

Given induction of FRY by macrophages, we examined its clinical significance using publicly available ovarian cancer datasets. Analysis of TCGA-OV (*n* = 315) and GSE9899 (*n* = 291) cohorts showed that FRY expression correlates with the clinical stage. These cohorts predominantly comprise high-grade serous carcinoma (HGSOC), the most common and aggressive ovarian cancer subtype. Consistent with this, analysis of the GSE63885 cohort (*n* = 101) showed that HGSOC comprises 72.3% of cases, with FRY showing a trend toward higher expression in HGSOC compared to other subtypes, though statistical significance was not reached due to limited sample sizes of non-HGSOC subtypes (Figure S1). In TCGA-OV, both stage III and IV tumors exhibited significantly higher FRY levels than stage I–II tumors, whereas in GSE9899, a significant increase was observed, primarily in stage IV tumors (Figure 1B). Kaplan–Meier survival analysis revealed that high FRY expression was significantly associated with reduced overall survival in patients with ovarian cancer (Figure 1C). These findings indicate that FRY expression is clinically associated with a poor prognosis in ovarian cancer.

### 3.2. FRY Is Required for Macrophage-Driven Ovarian Cancer Cell Invasion

**Figure 2:**
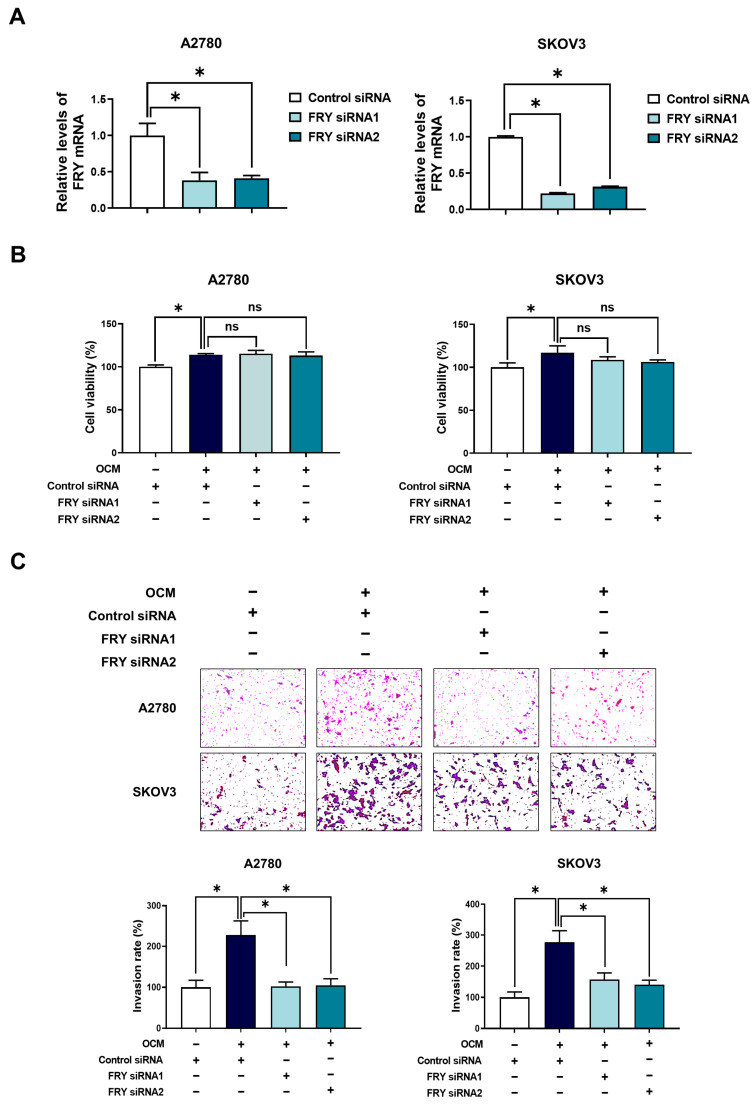
FRY knockdown reduces OCM-driven invasion in ovarian cancer cells. Cells were transfected with FRY siRNAs (10 nM, 24 h) or control siRNAs following OCM stimulation for 24 h. (**A**) RT-PCR confirming efficient FRY knockdown. (**B**) Cell viability assessment showing that FRY knockdown does not alter viability in OCM-treated cells. (**C**) Invasion assay showing that OCM increases invasion, while FRY knockdown significantly reduces this OCM-induced invasive response. * *p* < 0.05; ns, not significant.

To determine whether FRY upregulation contributed to macrophage-induced ovarian cancer cell invasion, we conducted functional studies using siRNA-mediated FRY knockdown. Knockdown efficiency in A2780 and SKOV3 cells was verified by RT-PCR, which demonstrated a substantial reduction in FRY mRNA levels (Figure 2A). FRY knockdown by FRY siRNA1 and FRY siRNA2 was further validated using two additional independent RT-PCR primer sets targeting different regions of the FRY transcript, which yielded consistent results (Figure S2). Importantly, FRY knockdown did not compromise cell viability under OCM stimulation, indicating that the observed effects on cell invasion were not due to cytotoxicity (Figure 2B). Invasion assays revealed that FRY knockdown substantially attenuated OCM-induced invasion of both ovarian cancer cell lines (Figure 2C). These results demonstrated that FRY plays a critical role in mediating macrophage-driven ovarian cancer cell invasion.

### 3.3. FRY Mediates OCM-Induced EMT Activation

**Figure 3:**
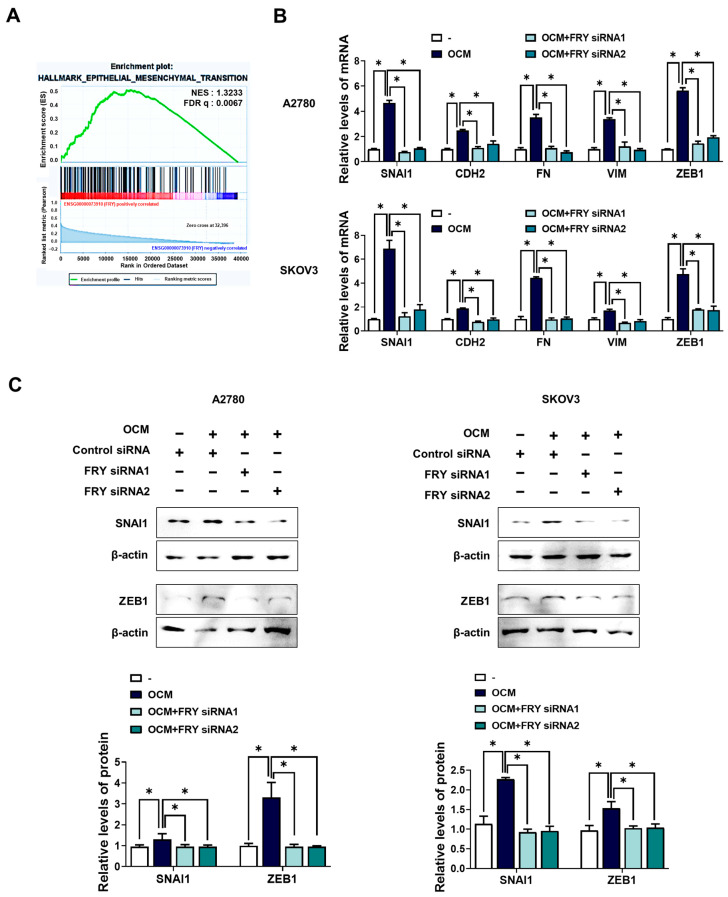
FRY is required for OCM–induced epithelial–mesenchymal transition (EMT) marker induction. (**A**) Gene Set Enrichment Analysis (GSEA) showing that ovarian cancer tumors with relatively high FRY expression (ENSG00000073910) exhibit significant enrichment of EMT-associated gene signatures. (**B**,**C**) Cells were transfected with FRY siRNAs (10 nM, 24 h) or control siRNAs following OCM stimulation for 24 h. (**B**) RT-PCR analysis of EMT markers (SNAI1, CDH2, FN, VIM, and ZEB1) after FRY knockdown. (**C**) Western blot analysis of SNAI1 and ZEB1 protein levels under FRY knockdown. * *p* < 0.05.

To explore the molecular mechanisms by which FRY promotes ovarian cancer cell invasion, we investigated its potential link to EMT, a critical process driving cancer metastasis. GSEA revealed a significant enrichment of EMT-related gene sets in ovarian cancer tumors exhibiting relatively high FRY expression (Figure 3A), suggesting a connection between FRY and EMT activation. To validate this association, we examined the expression of mesenchymal markers in ovarian cancer cells exposed to OCM. RT-PCR analysis demonstrated that OCM substantially increased the mRNA levels of key mesenchymal markers including SNAI1, N-cadherin (CDH2), fibronectin (FN), vimentin (VIM), and ZEB1 in both A2780 and SKOV3 cells (Figure 3B). Western blot analysis confirmed the elevated protein levels of SNAI1 and ZEB1 following OCM treatment (Figure 3C).

To determine whether FRY mediates OCM-induced EMT, we performed FRY knockdown experiments. Notably, FRY knockdown using specific siRNAs markedly attenuated the OCM-induced upregulation of all examined EMT markers at both the mRNA (Figure 3B) and protein (Figure 3C) levels. In parallel, we observed a reciprocal change in the epithelial marker E-cadherin: OCM reduced E-cadherin expression, whereas FRY knockdown reversed this reduction and restored E-cadherin toward baseline levels (Figure S3). Together, the reciprocal regulation of epithelial (E-cadherin) and mesenchymal markers supports the hypothesis that FRY functionally governs the EMT program rather than modulating individual markers in isolation. These findings indicate that FRY is required for OCM-mediated EMT activation and suggest that FRY promotes mesenchymal transition, contributing to the invasive phenotype of ovarian cancer cells.

### 3.4. FRY Functions Downstream of PI3K/AKT Signaling

**Figure 4:**
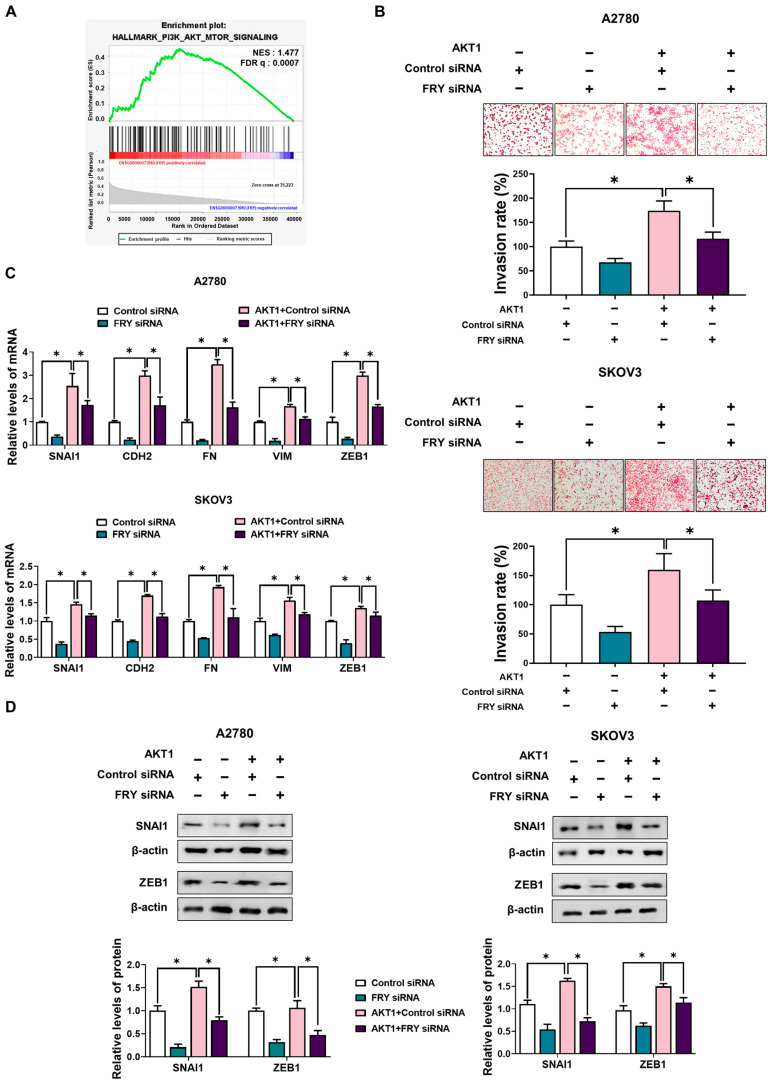
FRY acts downstream of PI3K/AKT signaling to mediate invasion. (**A**) GSEA enrichment plot showing significant association between the PI3K/AKT/mTOR pathway signatures and high FRY expression (ENSG00000073910) in ovarian cancer tissues. (**B**–**D**) Cells were transfected with AKT1 overexpression plasmid (10 nM) and FRY siRNA (10 nM), and followed by treatment with OCM for 24 h. (**B**) Invasion assay showing that FRY knockdown rescues the invasive phenotype induced by AKT1 overexpression. (**C**) RT-PCR analysis of EMT marker genes. (**D**) Western blot showing reduced mesenchymal marker expression after FRY knockdown in AKT1-overexpressing, OCM-treated cells. * *p* < 0.05.

To delineate the signaling pathways through which FRY promotes cell invasion, we analyzed the transcriptomic signatures associated with FRY expression. GSEA revealed significant enrichment of the PI3K/AKT/mTOR pathway signatures in tumors exhibiting FRY expression (Figure 4A). Given that the PI3K/AKT axis is a well-established driver of ovarian cancer metastasis [20,21], we hypothesized that FRY functions as a key effector within this cascade. To test this hypothesis, we assessed whether FRY was necessary for AKT activation. While AKT1 overexpression robustly enhanced cell invasion, this invasive phenotype was significantly abrogated by concurrent FRY knockdown (Figure 4B). This suggests that FRY is not merely correlated with but is functionally required for AKT1-mediated invasion. Furthermore, RT-PCR analysis showed that AKT1-induced upregulation of key EMT markers (SNAI1, CDH2, FN1, VIM, and ZEB1) was markedly attenuated by FRY downregulation (Figure 4C). These results were corroborated at the protein level, with Western blotting confirmed that FRY knockdown suppressed AKT1-driven expression of mesenchymal proteins in OCM-treated cells (Figure 4D). Collectively, these data suggest that FRY is an essential downstream mediator linking PI3K/AKT signaling to EMT and invasion in the context of the TME.

### 3.5. Nuclear Factor IX (NFIX) Regulates Macrophage-Induced FRY Expression

**Figure 5:**
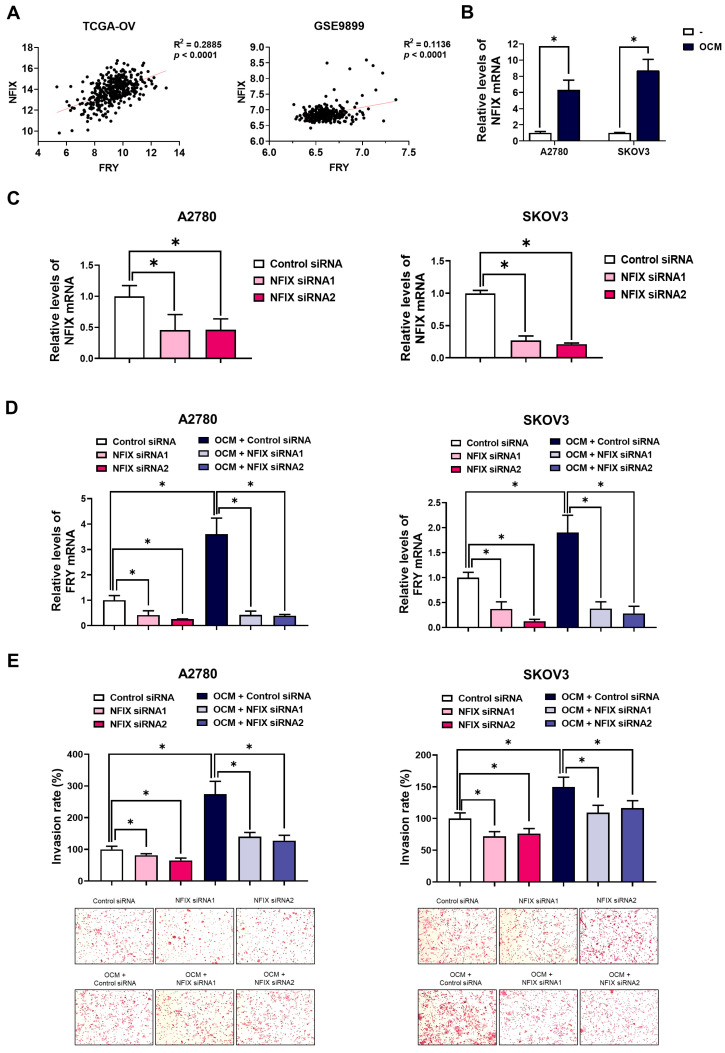
NFIX regulates FRY and OCM-induced invasion. (**A**) Pearson correlation analysis of NFIX and FRY expression in TCGA-OV (*n* = 379) and GSE9899 (*n* = 291) cohorts. (**B**) RT-PCR analysis showing NFIX induction after 24 h OCM treatment. (**C**–**E**) Cells were transfected with control or NFIX siRNAs (10 nM, 24 h) and subsequently treated with OCM for 24 h. (**C**) RT-PCR confirming NFIX knockdown. (**D**) Effect of NFIX knockdown on basal and OCM-induced FRY expression. (**E**) Invasion assay demonstrating reduced invasiveness upon NFIX silencing. * *p* < 0.05.

We identified the upstream transcriptional machinery governing FRY upregulation in response to macrophage signaling. We focused on nuclear factor IX (NFIX), a transcription factor implicated in the invasive progression of ovarian cancer [22]. Analysis of clinical datasets revealed a significant positive correlation between FRY and NFIX mRNA levels in both TCGA-OV (R^2^ = 0.2885, *p* < 0.0001) and GSE9899 (R^2^ = 0.1136, *p* < 0.0001) cohorts (Figure 5A). To determine whether NFIX responded to macrophage-derived cues, we treated ovarian cancer cells with OCM. This stimulation resulted in marked upregulation of NFIX mRNA in both A2780 and SKOV3 cells (Figure 5B). To link NFIX to FRY regulation, we examined the effect of NFIX knockdown on OCM-induced FRY expression and invasion of ovarian cancer cells. NFIX knockdown not only efficiently downregulated NFIX levels (Figure 5C) but also significantly inhibited FRY expression under both basal and OCM-stimulated conditions (Figure 5D). Functionally, NFIX silencing recapitulated the FRY knockdown phenotype and substantially suppressed OCM-induced invasion (Figure 5E). These findings identify NFIX as a requisite transcriptional regulator that bridges macrophage signaling with FRY expression and subsequent invasion.

### 3.6. C-X-C Motif Chemokine Receptor (CXCR)1/2 Inhibition Suppresses Macrophage-Stimulated FRY Induction

**Figure 6:**
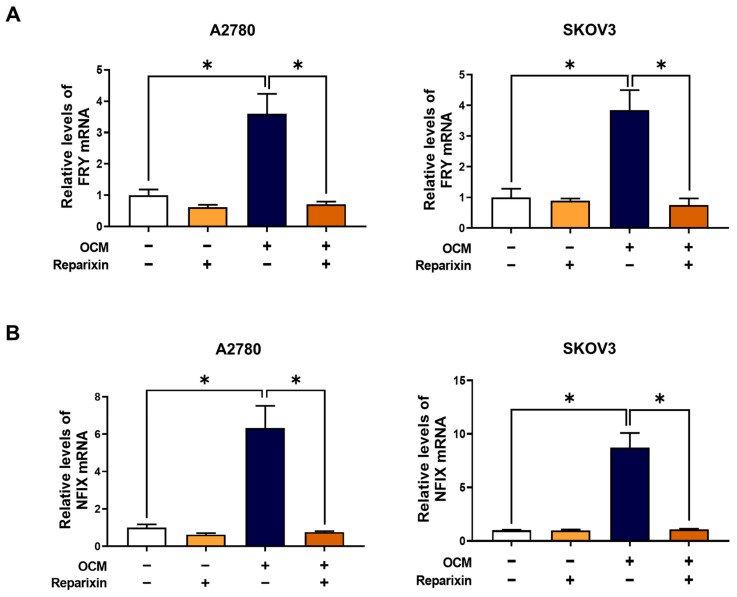
Pharmacological inhibition of CXCR1/2 blocks the macrophage-induced NFIX-FRY axis. Cells were pretreated with the CXCR1/2 inhibitor reparixin (0.1 μM, 2 h) and subsequently stimulated with OCM for 24 h. RT-PCR analysis of (**A**) FRY and (**B**) NFIX mRNA levels demonstrates that CXCR1/2 blockade prevents the OCM-mediated induction of both genes. * *p* < 0.05.

Macrophage-driven aggression in ovarian cancer is frequently mediated by chemokine networks, particularly the CXCR1/2 axis [17,23]. Building on our previous finding that macrophage-derived CXCL8 promotes invasion via CXCR1/2 [12], we investigated whether this pathway lies upstream of the NFIX–FRY axis (Figure 6). To test this hypothesis, we used reparixin, a specific inhibitor of CXCR1/2. Reparixin pretreatment consistently suppressed OCM-induced upregulation of FRY in both A2780 and SKOV3 cells (Figure 6A), indicating that CXCR1/2 activity is required for macrophage-mediated FRY induction. While reparixin treatment resulted in a modest, cell line-dependent reduction in basal FRY levels, it consistently and potently blocked OCM-induced upregulation of FRY in both A2780 and SKOV3 cells. Importantly, reparixin exerted a similar inhibitory effect on NFIX expression (Figure 6B), mirroring the pattern observed following FRY treatment. These data indicate that the CXCR1/2 signaling axis serves as the primary conduit for macrophage-to-tumor communication, initiating NFIX-mediated induction of FRY.

## 4. Discussion

We employed PMA-differentiated THP1 cells as an initial macrophage-like platform because their standardized differentiation and reduced donor-to-donor variability enable consistent mechanistic dissection of tumor-conditioned macrophage signaling. Nevertheless, we acknowledge that a monocytic cell line–derived model cannot fully capture the breadth of primary macrophage programs and TAM heterogeneity observed in human ovarian tumors. To mitigate this limitation and to provide patient-level support for the proposed macrophage–FRY relationship, we interrogated the TCGA-OV cohort using CIBERSORTx digital cytometry and observed that tumors with high FRY mRNA expression exhibit a significantly increased proportion of M2-like macrophages (Figure S4). This finding aligns with our experimental results showing that ovarian cancer–stimulated macrophage-derived signals induce FRY expression, and it supports the notion that FRY-high tumors may preferentially arise within M2-rich microenvironments where persistent macrophage signaling contributes to FRY upregulation and downstream tumor-promoting phenotypes. Importantly, we view this analysis as supportive rather than definitive, because immune deconvolution from bulk transcriptomes cannot establish causality or resolve macrophage functional states at single-cell resolution. Accordingly, follow-up work will prioritize experimental validation using PBMC-derived macrophages and macrophages from preclinical ovarian cancer models, and will incorporate approaches that better resolve TAM diversity beyond the M1/M2 framework.

FRY is primarily characterized as a MAP involved in morphogenesis, cytoskeletal organization, and cellular architecture [24,25]. These functions place FRY among the proteins that contribute to coordinated changes in cell shape, polarity, and structural stability. A recent study reported that alterations in FRY expression can influence proliferative and morphological behaviors in breast cancer models [11], suggesting that FRY participates in broader cytoskeleton-linked pathways relevant to malignant progression. Despite these findings, the pathological significance of FRY in ovarian cancer, particularly its potential involvement in invasion, has not yet been explored.

EMT is a key driver of ovarian cancer dissemination and is tightly coupled with cytoskeletal remodeling [26]. MAPs play important roles in these processes; for example, the microtubule end-binding protein EB1 has been implicated in promoting migratory and invasive phenotypes in cancer cells [10] and multiple MAPs have been linked to metastatic behavior through the modulation of microtubule dynamics and cellular organization [27]. Therefore, the association between FRY and microtubules provides a biologically plausible context for their involvement in EMT-related invasive states. The observation that EMT-related gene signatures clustered with relatively higher FRY expression in ovarian tumors, together with the reversal of EMT marker induction upon FRY reduction, supports the idea that FRY may influence epithelial–mesenchymal plasticity through its cytoskeletal functions.

The PI3K/AKT pathway is a major driver of ovarian cancer progression and is strongly activated by cell–cell interactions in the TME [28,29,30]. Within this framework, FRY aligned with transcriptional programs associated with PI3K/AKT signaling in tumors, suggesting that FRY participates in AKT-linked invasive states. Because AKT promotes cytoskeletal remodeling and EMT-associated transcriptional outputs [26,30], the placement of a MAP, such as FRY, downstream of this pathway provides a mechanistic connection between cytoskeletal regulation and AKT-driven invasion. In particular, AKT can influence microtubule stability and organization via the PI3K–AKT–GSK3 axis, and PI3K/AKT signaling also interfaces with Rho-family GTPase–driven cytoskeletal remodeling to support cell motility and invasion [31,32]. Given that FRY has been described as a microtubule-associated protein, our finding that FRY is required for AKT-driven invasion is consistent with a model in which FRY functions as a cytoskeletal effector downstream of PI3K/AKT, although the precise biochemical linkage remains an important subject for future investigation.

NFIX has been implicated in transcriptional programs governing migration, EMT, and developmental processes [33,34], and is associated with invasive and stemness-related phenotypes in ovarian cancer [22]. Its close relationship with FRY expression across ovarian cancer datasets suggests its regulatory influence in maintaining both basal and inducible FRY transcription. Given that AKT activates diverse transcription factors, including those that shape the EMT, it is plausible that NFIX participates in AKT-responsive regulatory networks. Although a direct AKT–NFIX connection has not been established, the presence of both inputs into FRY supports a model in which FRY integrates multiple transcriptional or signaling pathways relevant to invasion.

CXCR1/2 signaling has been widely implicated in promoting aggressive phenotypes in ovarian cancer, including PI3K/AKT pathway activation, enhanced proliferation, migration, and therapeutic resistance [28,29]. Consistent with these reported functions, pharmacological inhibition of CXCR1/2 reduced OCM-induced increases in FRY expression, indicating that this receptor axis contributes to the regulation of these factors under macrophage-stimulated conditions. CXCR1/2 can be activated by multiple CXC chemokines (e.g., CXCL8, CXCL1, CXCL2, CXCL5, CXCL6), several of which are reported to be abundant in tumor-associated macrophage secretomes [35,36]. In our prior work, CXCL8 was highly enriched in patient-derived ascites macrophages and CXCL8–CXCR1/2 signaling regulated invasion-associated gene programs in ovarian cancer cells [12]. In our prior work, CXCL8 was highly enriched in patient-derived ascites macrophages and CXCL8–CXCR1/2 signaling regulated invasion-associated gene programs in ovarian cancer cells. Together, these observations support the possibility that CXCL8 and/or other CXCR1/2 ligands represent primary secreted inducers of FRY; comprehensive secretome profiling will be needed to define the full set of contributing macrophage-derived factors. Although the downstream signaling mechanisms linking CXCR1/2 activation to transcriptional control remain to be defined, these findings suggest that CXCR1/2 operates upstream of NFIX and FRY within the macrophage-influenced signaling networks in ovarian cancer.

A limitation of this study is that we could not directly quantify residual FRY protein levels after siRNA treatment. Accordingly, our conclusions are based on robust transcript-level knockdown validation and concordant phenotypes observed with two independent siRNAs across two ovarian cancer cell lines and multiple functional readouts; protein-level quantification and mechanistic validation of FRY regulation will be prioritized in future studies using optimized detection strategies.

## 5. Conclusions

FRY has emerged as a macrophage-responsive regulator of ovarian cancer invasion, acting through EMT induction and functioning downstream of PI3K/AKT while being regulated by NFIX. Its strong association with advanced clinical stages and reduced patient survival underscores its biological and clinical relevance. Together, these findings indicate that FRY participates in a network of microenvironment-driven pro-invasive pathways and highlight its potential as a therapeutic target for ovarian cancer.

## Data Availability

The original contributions presented in this study are included in the article/Supplementary Materials. Further inquiries can be directed to the corresponding author(s).

## Supplementary Materials

The following supporting information can be downloaded at: https://www.mdpi.com/article/10.3390/cells15030289/s1, Table S1: Oligonucleotide sequences used in this study, Figure S1: FRY transcript levels across histological subtypes in the GSE63885 cohort, Figure S2: Validation of FRY knockdown using independent RT-PCR primer sets, Figure S3: FRY mediates OCM-induced E-cadherin downregulation, Figure S4: Association between FRY expression and macrophage composition in ovarian cancer cohorts.

## Abbreviations

The following abbreviations are used in this manuscript:

TME	Tumor microenvironment
EMT	Epithelial–mesenchymal transition
MAPs	Microtubule-associated proteins
OCM	Conditioned medium from ovarian cancer-stimulated macrophages
GSEA	Gene Set Enrichment Analysis
HGSOC	High-grade serous carcinoma
NFIX	Nuclear factor IX
CXCR	C-X-C Motif Chemokine Receptor
