# Supplementary material for: FRY Mediates THP1-Driven Ovarian Cancer Invasion Through the PI3K/AKT Pathway
Source: Cells. 2026 Feb 3;15(3):289. doi: 10.3390/cells15030289 (PMC12896515; doi:10.3390/cells15030289)
Supplement: Supplementary file 1 [file cells-15-00289-s001.zip › cells-4090268_Supplementary figures.pdf]

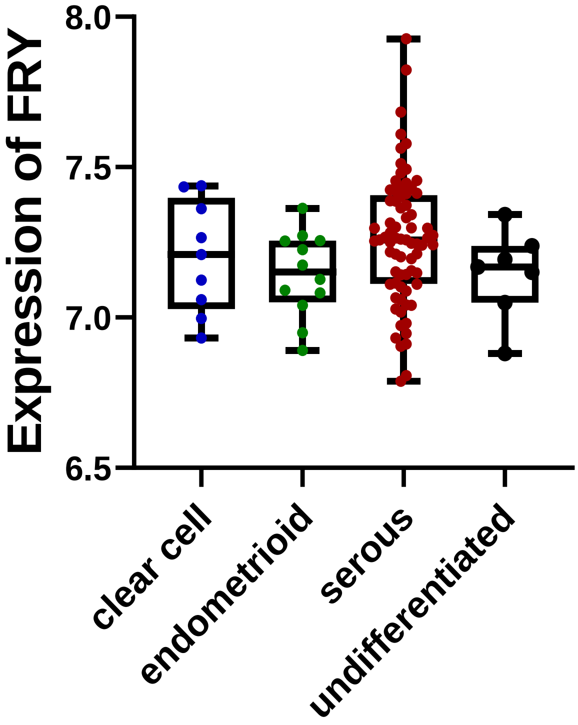

Supplementary Figure S1. FRY transcript levels across histological subtypes of ovarian cancer in the GSE63885 cohort. FRY mRNA expression is shown for clear cell (n=9), endometrioid (n=12), serous (HGSOC; n=73), and undifferentiated (n=7) ovarian cancers.

A

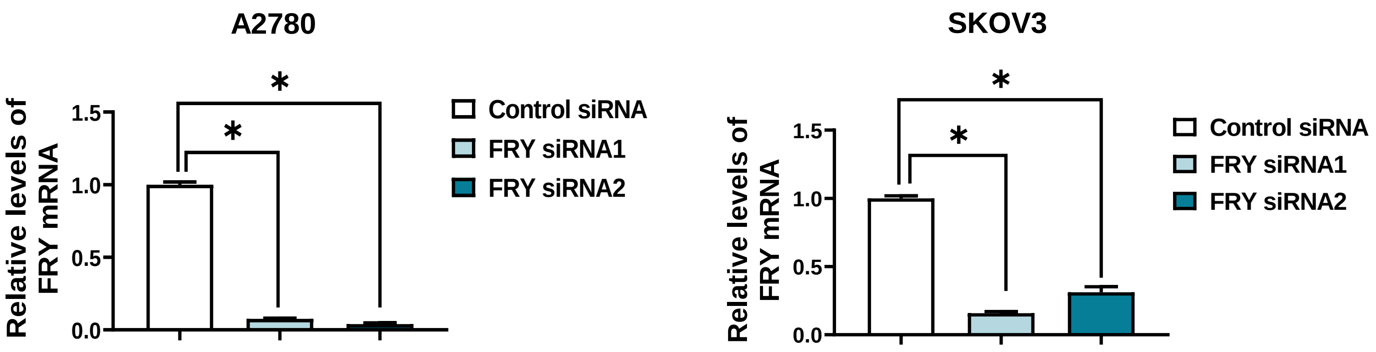

B

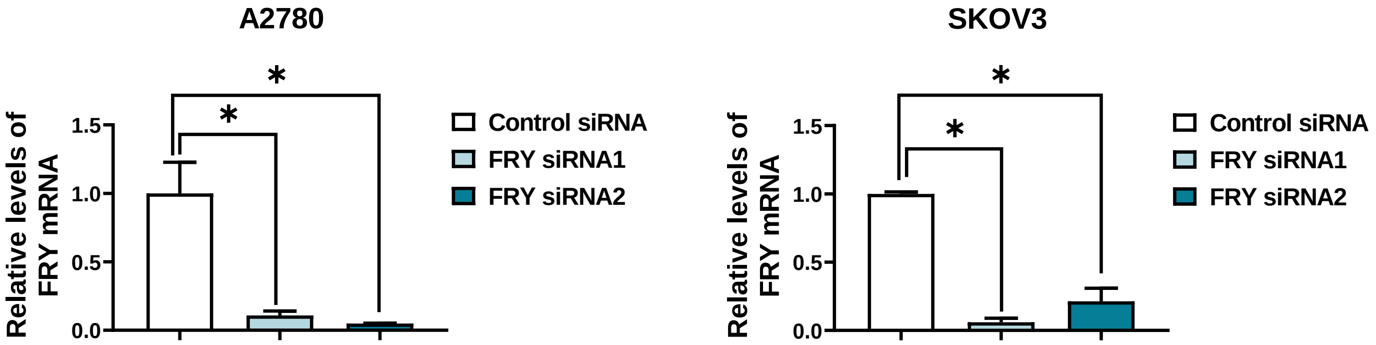

Supplementary Figure S2. Validation of FRY knockdown using independent RT-PCR primer sets.

FRY mRNA levels in A2780 and SKOV3 cells transfected with control siRNA, FRY siRNA #1, or FRY siRNA #2 were measured by RT-PCR using two newly designed independent primer sets targeting different regions of the FRY transcript. (**A**) Primer set #2. (**B**) Primer set #3. Both primer sets consistently confirmed efficient FRY knockdown by FRY siRNA1 and FRY siRNA2. These results are consistent with those obtained using the original primer set shown in Figure 2A. *$p < 0.05$.

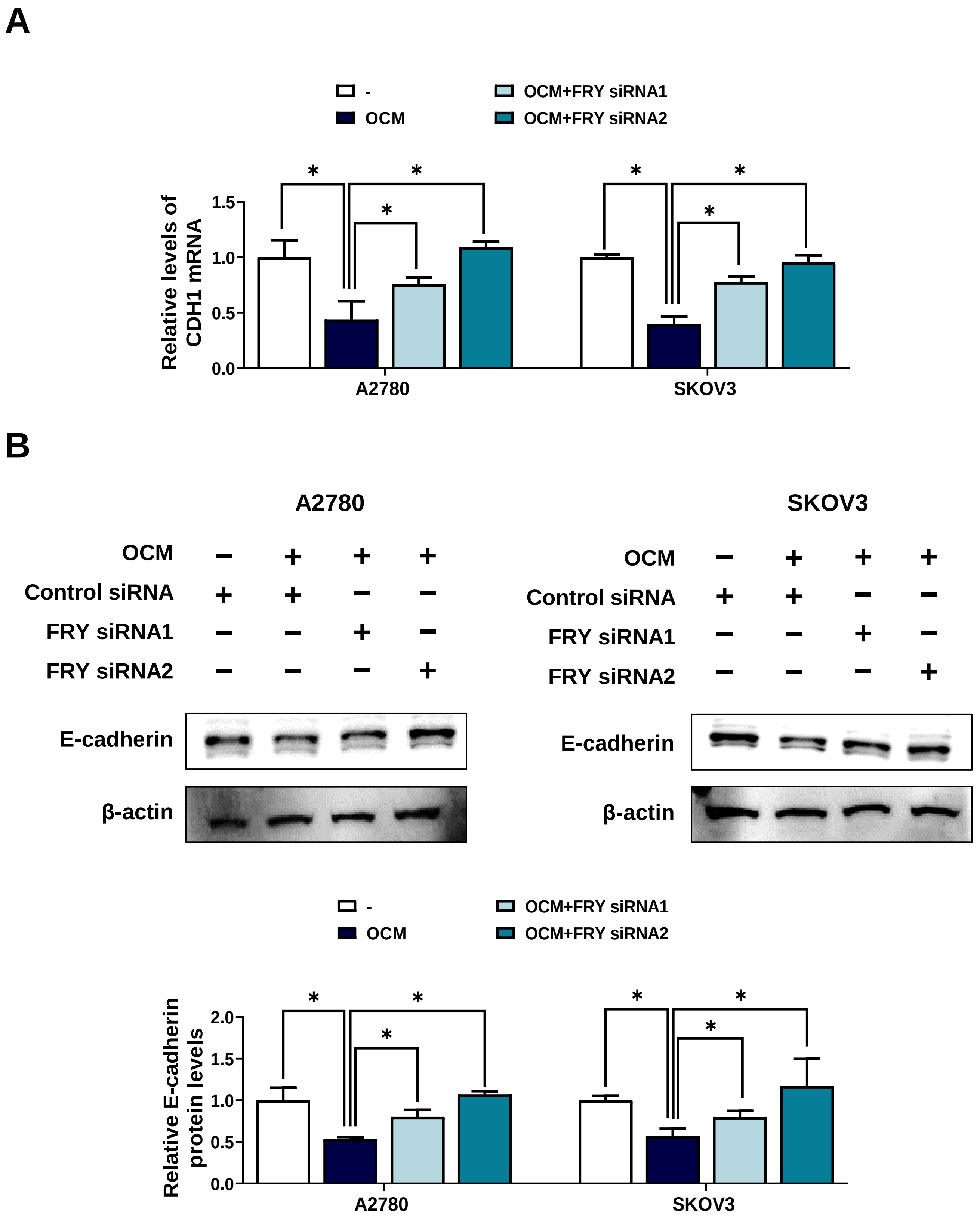

Supplementary Figure S3. FRY mediates OCM-induced E-cadherin downregulation.

Cells were transfected with FRY siRNAs (10 nM, 24 h) or control siRNAs following ovarian cancer macrophage–conditioned medium (OCM) stimulation for 24 h. (**A**) RT-PCR analysis of CDH1 mRNA after FRY knockdown. (**B**) Western blot analysis of E-cadherin protein levels under FRY knockdown. *$p < 0.05$.

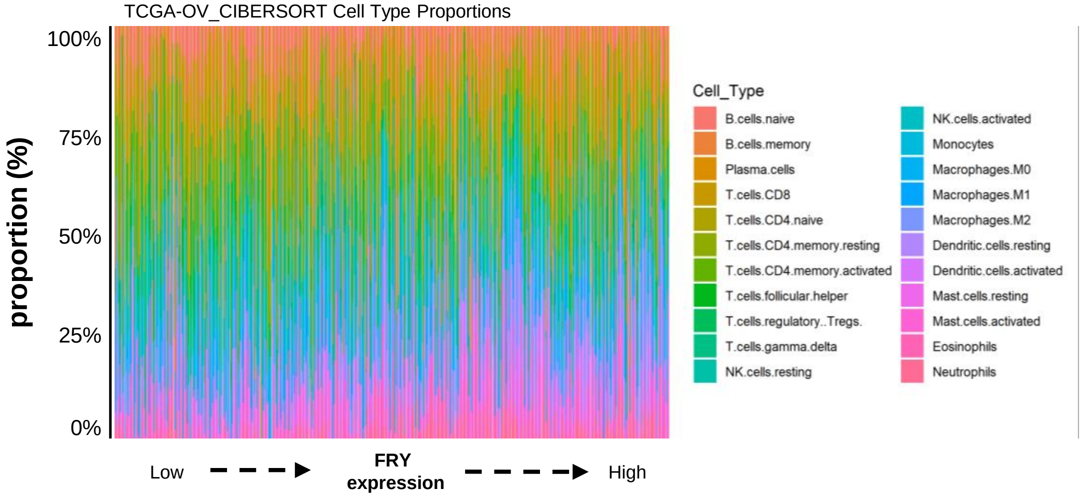

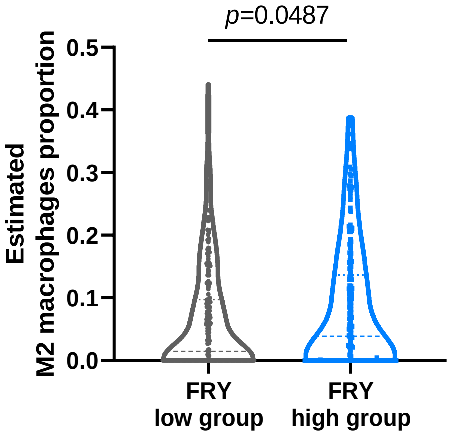

Supplementary Figure S4. Association between FRY expression and macrophage composition in ovarian cancer cohorts. Bulk RNA-seq data (TCGA-OV cohort, n=489) were analyzed using CIBERSORTx to estimate immune cell composition. For each cohort, the left panel shows the overall immune cell-type composition, and the right panel displays the estimated proportion of M2 macrophages in patients with high versus low FRY mRNA expression in tumor tissues. Tumors were stratified into FRY-low and FRY-high groups based on median FRY expression.
